# Value of individual surgeon performance metrics as quality assurance measures in oesophagogastric cancer surgery

**DOI:** 10.1002/bjs5.50230

**Published:** 2019-11-04

**Authors:** A. G. M. T. Powell, J. Wheat, N. Patel, D. Chan, A. Foliaki, S. A. Roberts, W. G. Lewis, on behalf of the South‐East Wales Oesophagogastric Cancer Collaborative, G. Blackshaw, G. Clark, A. Christian, X. Escofet, A. Foliaki, T. Havard, M. Henwood, J. Witherspoon, W. G. Lewis

**Affiliations:** ^1^ Division of Cancer and Genetics Cardiff University Cardiff UK; ^2^ Department of Surgery University Hospital of Wales Cardiff UK; ^3^ Department of Radiology University Hospital of Wales Cardiff UK

## Abstract

**Background:**

Surgeon‐level operative mortality is widely seen as a measure of quality after gastric and oesophageal resection. This study aimed to evaluate this alongside a compound‐level outcome analysis.

**Methods:**

Consecutive patients who underwent treatment including surgery delivered by a multidisciplinary team, which included seven specialist surgeons, were studied. The primary outcome was death within 30 days of surgery; secondary outcomes were anastomotic leak, Clavien–Dindo morbidity score, lymph node harvest, circumferential resection margin (CRM) status, disease‐free (DFS), and overall (OS) survival.

**Results:**

The median number of annual resections per surgeon was 10 (range 5–25), compared with 14 (5–25) for joint consultant teams (*P* = 0·855). The median annual surgeon‐level mortality rate was 0 (0–9) per cent *versus* an overall network annual operative mortality rate of 1·8 (0–3·7) per cent. Joint consultant team procedures were associated with fewer operative deaths (0·5 per cent *versus* 3·4 per cent at surgeon level; *P* = 0·027). The median surgeon anastomotic leak rate was 12·4 (range 9–20) per cent (*P* = 0·625 *versus* the whole surgical range), overall morbidity 46·5 (31–60) per cent (*P* = 0·066), lymph node harvest 16 (9–29) (*P* < 0·001), CRM positivity 32·0 (16–46) per cent (*P* = 0·003), 5‐year DFS rate 44·8 (29–60) per cent and OS rate 46·5 (35–53) per cent. No designated metrics were independently associated with DFS or OS in multivariable analysis.

**Conclusion:**

Annual surgeon‐level metrics demonstrated wide variations (fivefold), but these performance metrics were not associated with survival.

## Graphical abstract

Annual surgeon‐level metrics demonstrated the greatest variation (fivefold) risking inappropriate target thresholds. Surgeon‐level performance metrics were not associated with survival, suggesting that compound‐level quality assurance provides better overall performance appraisal.

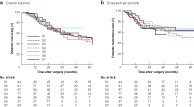

Annual figures can be misleading

## Abstract (es)

**Antecedentes:**

La mortalidad operatoria relacionada con el nivel del cirujano se contempla ampliamente como una medida de calidad tras la resección esofágica. Este estudio tenía como objetivo evaluar este aspecto junto con un análisis de resultados conjuntos a nivel de procedimientos.

**Métodos:**

Se estudiaron los pacientes consecutivos que fueron tratados, incluyendo el tratamiento quirúrgico, efectuado por un equipo multidisciplinar formado por siete cirujanos especialistas. La variable principal de resultados era la mortalidad a durante los primeros 30 días de la cirugía, y las variables secundarias fueron la fuga anastomótica, la gravedad de la puntuación de morbilidad de Clavien‐Dindo, el número de ganglios linfáticos obtenidos, el estado del margen circunferencial (*circumferential margin*, CRM), la supervivencia libre de enfermedad (*disease‐free survival*, DFS) y la supervivencia global (*overall survival*, OS).

**Resultados:**

La mediana del número anual de resecciones por cirujano fue de 10 (rango 5‐25, *P* = 0,855). El nivel de la mediana de mortalidad anual por cirujano fue del 0% (0‐9,1) y la mortalidad operatoria anual global del equipo de 1,8% (0‐3,7, P = 0,389). Los procedimientos conjuntos del equipo consultor se asociaron con menos muertes operatorias (0,5 versus 3,4%, *P* = 0,027). La tasa mediana (rango) de fuga anastomótica por cirujano fue del 12% (9‐20, *P* = 0,625), la morbilidad global del 46,7% (31‐60, *P* = 0,003), la DFS a los 5 años del 44,8% (28,6‐60,0, *P* = 0,257) y la OS del 46,5% (35,0‐52,5, *P* = 0,573). Ningún factor mostró una asociación independiente con la DFS o la OS en el análisis multivariable.

**Conclusión:**

Las medidas anuales a nivel de cirujano demostraron amplias variaciones (9 veces), pero estas medidas de rendimiento no se asociaron con la supervivencia.

## Introduction

There is considerable evidence that there is a relationship between increasing volume and decreasing surgical mortality following oesophagogastric resection for cancer. The evidence is stronger for institutional outcomes than for individual surgeons^1^, ^2^, ^3^. Specialist multidisciplinary team (MDT) expertise has been reported to improve patient outcomes^4^, ^5^, ^6^, but remains untested by a randomized trial.

Quality assurance metrics in surgery have traditionally included operative mortality within 30 days of an operation. The UK National Oesophago‐Gastric Cancer Audit includes other variables as indicators of surgical quality related to lymph node harvest, circumferential resection margin (CRM) involvement and duration of hospital stay, although not disease‐free (DFS) or overall (OS) survival.

The aim of this study was to evaluate all of the compound metrics of surgical quality assurance at surgeon and unit level, using time frames of 1‐, 3‐ and 5‐year survival as end‐points.

## Methods

The South East Wales Cancer Network serves a population of about 1·75 million. It involves ten acute, eight district general and two teaching hospitals. Before August 2010, eight surgeons undertook surgery at four different hospital sites, but since 2010 all resectional surgery has been undertaken at a single site involving six specialist upper gastrointestinal (UGI) surgeons carrying out all cancer resectional surgery, three based at the surgical centre and three operating on an in‐reach basis, with a facility for joint consultant operating. A seventh surgeon joined the team in 2017.

Diagnosis and staging was undertaken locally, coordinated via three local weekly MDT meetings. All patients deemed suitable for curative treatment were discussed at a weekly regional network MDT meeting. Integral to the new surgical model was the establishment of an enhanced recovery programme^7^ based on established principles in colorectal surgery^8^.

Data regarding the oesophageal and gastric cancer workload were collected using a combination of a prospectively developed database in combination with MDT records and review of hospital records. Pathological variables were recorded from histopathology reports issued at the time of surgery. CRM status was defined using the Royal College of Pathologists guidelines^9^, ^10^. Measures of outcome included postoperative morbidity and mortality, length of hospital stay and survival at 1, 3 and 5 years from diagnosis. Patients were followed up at regular intervals of 3 months for the first year and 6 months thereafter. In the event that patients developed symptoms suggestive of recurrent disease, investigations were undertaken sooner. Follow‐up surveillance was conducted for 5 years or until death, whichever was sooner. Dates and causes of death were obtained from the Wales Cancer Intelligence and Surveillance Unit and from the Office for National Statistics. Regional ethical approval was sought, but deemed unnecessary because the study was considered to be service evaluation.

All patients had management plans individually tailored according to factors relating to both patient and stage of disease^11^. Staging included CT, endoscopic ultrasonography, CT–PET and staging laparoscopy as appropriate. The South East Wales MDT treatment algorithms for oesophageal and gastric cancer have been described previously^12^, ^13^. Operative morbidity was graded in accordance with the Clavien–Dindo classification^14^, ^15^. Particular emphasis was placed on the incidence of morbidity of grade III or higher. Definitive chemoradiotherapy was offered to patients with localized squamous cell carcinoma and to patients with adenocarcinoma deemed unsuitable for surgery because of disease extent and/or medical co‐morbidity^16^, ^17^.

### Statistical analysis

Grouped data were expressed as median (i.q.r.) values, and non‐parametric statistical methods were used. Continuous data were compared using the Mann–Whitney *U* test, and categorical data using the χ^2^ test or Fisher's exact test when the number of events was low. A non‐parametric two‐sample test on the equality of medians was carried out. Differences were deemed to be statistically significant when the *P* value was less than 0·050.

DFS for all patients was calculated by measuring the interval from a landmark time of 6 months after diagnosis to the date of recurrence. This approach has been adopted in previous randomized trials^18^, to allow for the variable interval to surgery following diagnosis, depending on whether neoadjuvant therapy was prescribed. Events resulting in a failure to complete curative treatment, such as not proceeding to surgery, open and close laparotomy, palliative resection, in‐hospital mortality and disease progression during neoadjuvant chemotherapy, were assumed to have occurred at this landmark time, to maintain the intention‐to‐treat analysis. Overall survival was measured from the date of diagnosis. Cumulative survival was calculated according to the Kaplan–Meier method; differences between groups were analysed with the log rank test. Proportional hazard plots were created and Schoenfeld residuals were calculated to confirm that the proportional hazard assumption was appropriate for overall survival. Univariable analyses involving potential factors influencing survival were examined initially by the life‐table method of Kaplan and Meier, and those with associations where *P* < 0·010 were retained in a Cox proportional hazards model using forward conditional methodology to assess the prognostic value of individual variables.

All statistical analysis was performed using IBM® SPSS® statistics v25.0.0.0 (IBM, Armonk, New York, USA) with extension R.

## Results

In total, 525 patients were identified who underwent surgery between August 2010 and August 2018 for oesophagogastric cancer (oesophageal, 311; gastric, 214), including 206 procedures (oesophageal cancer, 120 (58·3 per cent); gastric cancer, 86 (41·7 per cent)) performed by two consultants working together. Sixteen patients had laparoscopically assisted surgery during the study period (oesophagectomy, 14 patients (5·3 per cent); gastrectomy, 2 (1·1 per cent)). The median annual resection number per surgeon was 10 (range 5–25), compared with 14 (5–25) for joint consultant teams (*P* = 0·855). The numbers of surgical procedures performed by each surgeon (S) and as a team (in parentheses) were: S1 64 (36), S2 92 (10), S3 112 (32), S4 90 (64), S5 54 (40), S6 38 (3) and S7 75 (21).

The median age of patients undergoing resection was 66 (i.q.r. 59–72) years; some 42·3 per cent were aged less than 65 years. Most patients were men (77·3 per cent), and had oesophageal cancer (59·2 per cent). For the whole cohort of 525 patients, neoadjuvant therapy was offered to 296 patients (56·4 per cent) (chemotherapy, 238 (45·3 per cent); chemoradiotherapy, 58 (11·0 per cent)). Some 244 patients (46·5 per cent) developed postoperative complications, of which 56 (12·4 per cent) were due to anastomotic leak. There were 12 deaths (2·3 per cent) within 30 days of surgery. The median length of hospital stay (LOS) for patients having a resection was 14 (range 11–20) days (*Table* 1).

**Table 1:** Collective and individual surgeon‐level postoperative outcome

	Collective (*n* = 525)	S1 (*n* = 64)	S2 (*n* = 92)	S3 (*n* = 112)	S4 (*n* = 90)	S5 (*n* = 54)	S6 (*n* = 38)	S7 (*n* = 75)
**Age (years)**								
< 65	222 (42·3)	23 (36)	40 (43)	57 (50·9)	30 (33)	26 (48)	13 (34)	33 (44)
65–75	217 (41·3)	21 (33)	36 (39)	41 (36·6)	42 (47)	23 (43)	21 (55)	33 (44)
> 75	86 (16·4)	20 (31)	16 (17)	14 (12·5)	18 (20)	5 (9)	4 (11)	9 (12)
**Sex**								
F	119 (22·7)	18 (28)	24 (26)	32 (28·6)	17 (19)	9 (17)	6 (16)	13 (17)
M	406 (77·3)	46 (72)	68 (74)	80 (71·4)	73 (81)	45 (83)	32 (84)	62 (83)
**Neoadjuvant therapy**†								
None	197 (43·8)	23 (52)	33 (41)	49 (51)	44 (57)	20 (44)	7 (19)	21 (30)
Chemotherapy	200 (44·4)	16 (36)	39 (48)	38 (39)	26 (34)	18 (40)	23 (62)	40 (58)
Chemoradiotherapy	53 (11·8)	5 (11)	9 (11)	10 (10)	7 (9)	7 (16)	7 (19)	8 (12)
**Tumour location**								
Oesophagus	311 (59·2)	33 (52)	55 (60)	69 (61·6)	44 (49)	34 (63)	26 (68)	50 (67)
Stomach	214 (40·8)	31 (48)	37 (40)	43 (38·4)	46 (51)	20 (37)	12 (32)	25 (33)
**Operation type†**								
Transhiatal oesophagectomy	140 (31·1)	20 (45)	23 (28)	44 (45)	23 (30)	15 (33)	2 (5)	13 (19)
Ivor Lewis procedure	126 (28·0)	5 (11)	27 (33)	15 (15)	14 (18)	12 (27)	20 (54)	33 (48)
Total gastrectomy	96 (21·3)	6 (14)	17 (21)	16 (16)	21 (27)	10 (22)	10 (27)	16 (23)
Subtotal gastrectomy	88 (19·6)	13 (30)	14 (17)	22 (23)	19 (25)	8 (18)	5 (14)	7 (10)
**Open and close**								
No	450 (85·7)	44 (69)	81 (88)	97 (86·6)	77 (86)	45 (83)	37 (97)	69 (92)
Yes	75 (14·3)	20 (31)	11 (12)	15 (13·4)	13 (14)	9 (17)	1 (3)	6 (8)
**Margin status†**								
Negative	306 (68·0)	34 (77)	44 (54)	66 (68)	65 (84)	33 (73)	21 (57)	43 (62)
Positive	144 (32·0)	10 (23)	37 (46)	31 (32)	12 (16)	12 (27)	16 (43)	26 (38)
Lymph node yield*	16 (11–23)	15 (9–21)	17 (13–24)	14 (10–18)	14 (10–20)	11 (9–20)	13 (11–20)	24 (17–29)
**Morbidity**								
No	280 (53·3)	44 (69)	49 (53)	61 (54·5)	49 (54)	27 (50)	20 (53)	30 (40)
Yes	245 (46·7)	20 (31)	43 (47)	51 (45·5)	41 (46)	27 (50)	18 (47)	45 (60)
**Anastomotic leak†**								
No	394 (87·6)	40 (91)	71 (88)	87 (90)	69 (90)	36 (80)	33 (89)	58 (84)
Yes	56 (12·4)	4 (9)	10 (12)	10 (10)	8 (10)	9 (20)	4 (11)	11 (16)
**Clavien–Dindo morbidity grade**								
0	255 (48·6)	42 (66)	42 (46)	57 (50·9)	43 (48)	24 (44)	19 (50)	28 (37)
I	25 (4·8)	2 (3)	7 (8)	4 (3·6)	6 (7)	3 (6)	1 (3)	2 (3)
II	147 (28·0)	14 (22)	24 (26)	29 (25·9)	25 (28)	13 (24)	9 (24)	33 (44)
III	61 (11·6)	4 (6)	13 (14)	13 (11·6)	9 (10)	9 (17)	3 (8)	10 (13)
IV	25 (4·8)	2 (3)	4 (4)	7 (6·3)	5 (6)	3 (6)	3 (8)	1 (1)
V	12 (2·3)	0 (0)	2 (2)	2 (1·8)	2 (2)	2 (4)	3 (8)	1 (1)
**30‐day mortality**								
No	513 (97·7)	64 (100)	90 (98)	110 (98·2)	88 (98)	52 (96)	35 (92)	74 (99)
Yes	12 (2·3)	0 (0)	2 (2)	2 (1·8)	2 (2)	2 (4)	3 (8)	1 (1)
Length of hospital stay (days)*	14 (11–20)	13 (11–15)	15 (12–21)	15 (12–21)	14 (12–23)	13 (11–18)	14 (11–18)	14 (12–18)

Values in parentheses are percentages unless indicated otherwise; *values are median (i.q.r.). †In the 450 patients who had a resection. S, surgeon.

During follow‐up, 122 patients (23·2 per cent) developed cancer recurrence, and 213 (40·6 per cent) died.

### Unit *versus* individual surgeon data

Comparative data reported for unit and surgeon outcome measures across the study period are shown in *Table* 1. Transhiatal oesophagectomy was the commonest procedure (140) (31·1 (range 5–45) per cent), followed by Ivor Lewis oesophagectomy (126) (28·0 (11–54) per cent), total gastrectomy (96) (21·3 (16–27) per cent) and subtotal gastrectomy (88) (19·6 (10–30) per cent). Open and close laparotomy was performed in 75 patients (14·3 (range 3–31·3) per cent).

The CRM was positive in 144 patients (32·0 per cent) (surgeon range 16–46 per cent) (*P* = 0·003 *versus* the whole surgical range) and median lymph node yield was 16 (i.q.r. 11–23) (surgeon range 11 (i.q.r. 9–20) to 24 (17–29)) (*P* < 0·001). Postoperative morbidity occurred in 245 patients (46·7 (range 31–60) per cent) (*P* = 0·066). Anastomotic leak occurred in 56 patients (12·4 (range 9–20) per cent) (*P* = 0·625). Major postoperative morbidity (grade III or above) was observed in 98 patients (18·7 (range 9–26) per cent) and median LOS was 14 (i.q.r. 11–20) days, similar for all surgeons. The operative 30‐day mortality rate was 2·3 per cent (surgeon range 0–8 per cent).

The 1‐, 3‐ and 5‐year survival rates at unit and individual level are shown in *Table* *S1* (supporting information). For oesophageal cancer, 1‐, 3‐ and 5‐year OS rates were 93·7 (range 86–100), 62·5 (54–71) and 44·6 (36–52) per cent respectively, with 1‐, 3‐ and 5‐year DFS rates of 79·3 (69–91), 50·0 (20–59) and 37·2 (25–56) per cent respectively. For gastric cancer, the 1‐, 3‐ and 5‐year OS rates were 89·6 (73–100), 61·8 (42–100) and 48·7 (33–56·5) per cent respectively, with 1‐, 3‐ and 5‐year DFS rates of 88·4 (69–100), 62·8 (40–80) and 57·4 (33–71) per cent respectively.

### Outcome parameters for consultant team approach

Baseline characteristics for patients grouped into individual consultant and dual team consultant operating are shown in *Table* 2. Dual consultant operating was performed in 206 procedures (39·2 per cent). Patients were younger in the dual consultant operator cohort compared with those in the individual consultant operator cohort (age below 65 years: 47·6 *versus* 38·9 per cent respectively; *P* = 0·029). Proportions were similar with regard to sex (22·8 *versus* 22·6 per cent; *P* = 0·986), neoadjuvant chemotherapy (46·1 *versus* 44·8 per cent; *P* = 0·394) and tumour location (58·3 *versus* 59·9 per cent; *P* = 0·712). Open and close operations were performed more commonly by dual consultant teams (18·0 per cent *versus* 11·9 for individual surgeons; *P* = 0·053), with lower lymph node harvests (median 14 *versus* 16 respectively; *P* = 0·012) and a lower operative mortality rate within 30 days of surgery (0·5 *versus* 3·4 per cent; *P* = 0·027). CRM positivity (28·4 *versus* 34·2 per cent; *P* = 0·205), postoperative morbidity (51·5 *versus* 52·7 per cent; *P* = 0·807), anastomotic leak (12·4 *versus* 12·5 per cent; *P* = 0·993) and grade 0 morbidity (43·2 *versus* 42·7 per cent; *P* = 0·467) were similar irrespective of operative team.

**Table 2:** Influence of dual consultant team operating strategy on clinical outcomes

	Team operating (*n* = 206)	Individual operating (*n* = 319)	*P* ‡
**Age (years)**			0·029
< 65	98 (47·6)	124 (38·9)	
65–75	81 (39·3)	136 (42·6)	
> 75	27 (13·1)	59 (18·5)	
**Sex**			0·986
F	47 (22·8)	72 (22·6)	
M	159 (77·2)	247 (77·4)	
**Neoadjuvant therapy**			0·394
None	93 (45·1)	136 (42·6)	
Chemotherapy	95 (46·1)	143 (44·8)	
Chemoradiotherapy	18 (8·7)	40 (12·5)	
**Tumour location**			0·712
Oesophagus	120 (58·3)	191 (59·9)	
Stomach	86 (41·7)	128 (40·1)	
**Operation type** †			0·505
Transhiatal oesophagectomy	63 (37·3)	77 (27·4)	
Ivor Lewis procedure	36 (21·3)	90 (32·0)	
Total gastrectomy	35 (20·7)	61 (21·7)	
Subtotal gastrectomy	35 (20·7)	53 (18·9)	
**Open and close**			0·053
No	169 (82·0)	281 (88·1)	
Yes	37 (18·0)	38 (11·9)	
**Margin status** †			0·205
Negative	121 (71·6)	185 (65·8)	
Positive	48 (28·4)	96 (34·2)	
**Lymph node yield** *, †	14 (10–21)	16 (11–24)	0·012§
**Morbidity** †			0·807
No	82 (48·5)	133 (47·3)	
Yes	87 (51·5)	148 (52·7)	
**Anastomotic leak** †			0·993
No	148 (87·6)	246 (87·5)	
Yes	21 (12·4)	35 (12·5)	
**Clavien–Dindo morbidity grade** †			0·467
0	73 (43·2)	120 (42·7)	
I	9 (5·3)	13 (4·6)	
II	54 (32·0)	85 (30·2)	
III	23 (13·6)	37 (13·2)	
IV	9 (5·3)	15 (5·3)	
V	1 (0·6)	11 (3·9)	
**30‐day mortality**			0·027
No	205 (99·5)	308 (96·6)	
Yes	1 (0·5)	11 (3·4)	
**Length of hospital stay** *	14 (12–20)	14 (11–20)	0·966§
**Overall survival (%)**			
1‐year	94·0	90·8	
2‐year	74·7	71·6	
3‐year	61·1	63·0	
5‐year	41·5	51·1	

Values in parentheses are percentages unless indicated otherwise;

* values are median (range).

† In the 450 patients who had a resection.

‡ χ^2^ or Fisher's exact test, except

§ Mann–Whitney *U* test.

### Collective, annual and 3‐year measures of surgical quality assurance

Complete characteristics related to quality assurance and outcome measures are shown in *Table* 3. Comparison of annual metrics in 2011 and 2018 revealed that in the latter year more patients received chemoradiotherapy (1 per cent in 2011 *versus* 14 per cent in 2018), more had an Ivor Lewis oesophagectomy (24 *versus* 41 per cent respectively) and fewer patients had an open and close procedure (17 *versus* 8 per cent). Other notable variations were found in the rates of open and close procedures (21 per cent in 2014 *versus* 8 per cent in 2018), lymph node yield (11 (i.q.r. 8–17) in 2014 *versus* 20 (15–27) in 2018), postoperative morbidity (26 per cent in 2013 *versus* 54 per cent in 2016), operative mortality (0 per cent in 2012 and 2014 *versus* 5 per cent in 2016) and 5‐year OS (55 per cent in 2014 *versus* 35 per cent in 2013) (*Table* 3). The median annual surgeon‐level mortality rate was 0 (0–9) per cent *versus* an overall network annual rate of 1·8 (0–3·7) per cent.

**Table 3:** Collective, 3‐yearly and annual measurements of operative and perioperative metrics

	Collective	3‐yearly measures			Yearly measures							
	2010–2018 (*n* = 525)	2010–2012 (*n* = 125)	2012–2015 (*n* = 177)	2015–2018 (*n* = 223)	2011 (*n* = 70)	2012 (*n* = 55)	2013 (*n* = 38)	2014 (*n* = 62)	2015 (*n* = 77)	2016 (*n* = 78)	2017 (*n* = 62)	2018 (*n* = 83)
**Neoadjuvant therapy**												
None	197 (43·8)	62 (49·6)	71 (40·1)	96 (43·0)	30 (43)	32 (58)	15 (39)	31 (50)	25 (32)	34 (44)	26 (42)	36 (43)
Chemotherapy	200 (44·4)	62 (49·6)	76 (42·9)	100 (44·8)	39 (56)	23 (42)	19 (50)	22 (35)	35 (45)	34 (44)	31 (50)	35 (42)
Chemoradiotherapy	53 (11·8)	1 (0·8)	30 (16·9)	27 (12·1)	1 (1)	0 (0)	4 (11)	9 (15)	17 (22)	10 (13)	5 (8)	12 (14)
**Operation type** †												
Transhiatal oesophagectomy	140 (31·1)	33 (30·8)	59 (39·6)	48 (24·7)	14 (24)	19 (39)	14 (41)	21 (43)	24 (36)	11 (17)	14 (26)	23 (30)
Ivor Lewis procedure	126 (28·0)	22 (20·6)	27 (18·1)	77 (39·7)	14 (24)	8 (16)	4 (12)	8 (16)	15 (23)	28 (43)	18 (34)	31 (41)
Total gastrectomy	96 (21·3)	25 (23·4)	33 (22·1)	38 (19·6)	15 (26)	10 (20)	8 (24)	10 (20)	15 (23)	17 (26)	9 (17)	12 (16)
Subtotal gastrectomy	88 (19·6)	27 (25·2)	30 (20·1)	31 (16·0)	15 (26)	12 (24)	8 (24)	10 (20)	12 (18)	9 (14)	12 (23)	10 (13)
**Open and close**												
No	450 (85·7)	107 (85·6)	149 (84·2)	194 (87·0)	58 (83)	49 (89)	34 (89)	49 (79)	66 (86)	65 (83)	53 (85)	76 (92)
Yes	75 (14·3)	18 (14·4)	28 (15·8)	29 (13·0)	12 (17)	6 (11)	4 (11)	13 (21)	11 (14)	13 (17)	9 (15)	7 (8)
**Margin status** †												
Negative	306 (68·0)	70 (65·4)	101 (67·8)	135 (69·6)	36 (62)	34 (69)	22 (65)	35 (71)	44 (67)	46 (71)	40 (75)	49 (64)
Positive	144 (32·0)	37 (34·6)	48 (32·2)	59 (30·4)	22 (38)	15 (31)	12 (35)	14 (29)	22 (33)	19 (29)	13 (25)	27 (36)
**Lymph node yield** *, †	16 (11–23)	16 (12–23)	12 (9–19)	17 (13–24)	17 (12–23)	15 (12–23)	12 (9–15)	11 (8–17)	14 (10–23)	14 (11–21)	17 (14–24)	20 (15–27)
**Morbidity** †												
No	280 (53·3)	69 (55·2)	107 (60·5)	104 (46·6)	41 (59)	28 (51)	28 (74)	37 (60)	42 (55)	36 (46)	29 (47)	39 (47)
Yes	245 (46·7)	56 (44·8)	70 (39·5)	119 (53·4)	29 (41)	27 (49)	10 (26)	25 (40)	35 (45)	42 (54)	33 (53)	44 (53)
**Anastomotic leak** †												
No	394 (87·6)	95 (88·8)	131 (87·9)	168 (86·6)	51 (88)	44 (90)	30 (88)	44 (90)	57 (86)	56 (86)	47 (89)	65 (86)
Yes	56 (12·4)	12 (11·2)	18 (12·1)	26 (13·4)	7 (12)	5 (10)	4 (12)	5 (10)	9 (14)	9 (14)	6 (11)	11 (14)
**Clavien–Dindo morbidity grade**												
0	255 (48·6)	66 (52·8)	90 (50·8)	99 (44·4)	38 (54)	28 (51)	18 (47)	34 (55)	38 (49)	35 (45)	26 (42)	38 (46)
I	25 (4·8)	3 (2·4)	17 (9·6)	5 (2·2)	3 (4)	0 (0)	10 (26)	3 (5)	4 (5)	1 | (1)	3 (5)	1 (1)
II	147 (28·0)	37 (29·6)	33 (18·6)	77 (34·5)	19 (27)	18 (33)	5 (13)	10 (16)	18 (24)	24 (31)	19 (31)	34 (41)
III	61 (11·6)	8 (6·4)	26 (14·7)	27 (12·1)	3 (4)	5 (9)	2 (5)	12 (19)	12 (16)	9 (12)	10 (16)	8 (10)
IV	25 (4·8)	9 (7·2)	8 (4·5)	8 (3·6)	5 (7)	4 (7)	1 (3)	3 (5)	4 (5.)	5 (6)	3 (5)	0 (0)
V	12 (2·3)	2 (1·6)	3 (1·7)	7 (3·1)	2 (3)	0 (0)	2 (5)	0 (0)	1 (1)	4 (5)	1 (2)	2 (2)
**30‐day mortality**												
No	513 (97·7)	123 (98·4)	174 (98·3)	216 (96·9)	68 (97)	55 (100)	36 (95)	62 (100)	76 (99)	74 (95)	61 (98)	81 (98)
Yes	12 (2·3)	2 (1·6)	3 (1·7)	7 (3·1)	2 (3)	0 (0)	2 (5)	0 (0)	1 (1)	4 (5)	1 (2)	2 (2)
**Length of hospital stay (days)** *, †	14 (11–20)	14 (11–19)	14 (11–20)	14 (12–20)	14 (11–20)	14 (11–18)	13 (12–17)	15 (13–23)	13 (11–19)	15 (13–25)	14 (12–23)	14 (11–16)
**Survival (%)**												
1‐year	92·1	92·5	89·3	94·0	91	94	82	92	91	92	94	98
2‐year	72·9	74·8	69·8	75·8	81	67	56	69	77	69	89	n.a.
3‐year	62·2	59·8	63·1	65·1	62	57	47	63	71	64	n.a.	n.a.
5‐year	46·5	47·8	47·7	44·4	50	45	35	55	n.a.	n.a.	n.a.	n.a.

Values in parentheses are percentages unless indicated otherwise;

* values are median (range).

† In the 450 patients who had a resection. n.a., Not available.

In contrast, when 3‐year time frames were examined, other than the prescription of neoadjuvant chemoradiotherapy (0·8 per cent in 2010–2012 *versus* 16·9 per cent in 2012–2015) and the proportion of patients having an Ivor Lewis oesophagectomy (18·1 per cent in 2012–2015 *versus* 39·7 per cent in 2015–2018), all other performance metrics were similar (*Table* 3).

### Survival analysis

**Figure 1:**
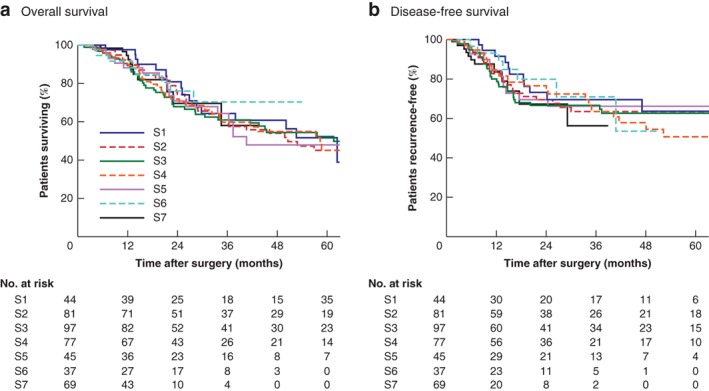
Kaplan–Meier analysis of overall and disease‐free survival, stratified by individual surgeon **a** Overall and **b** disease‐free survival. S, surgeon. **a** *P* = 0·993, **b** *P* = 0·930 (log rank test).

Univariable and multivariable survival analyses relating to all patients are shown in *Tables* 4, 5, 6. There was no relationship between OS or DFS and operating surgeon (*Fig*. 1).

**Table 4:** Univariable and multivariable analysis of clinicopathological factors and complication markers, overall and disease‐free survival in the whole cohort

	Overall survival				Disease‐free survival			
	Univariable analysis		Multivariable analysis		Univariable analysis		Multivariable analysis	
	Hazard ratio	*P*	Hazard ratio	*P*	Hazard ratio	*P*	Hazard ratio	*P*
**Age (< 65 *versus* 65–75 *versus* > 75 years)**	1·16 (0·94, 1·43)	0·176			0·85 (0·66, 1·10)	0·210		
**Sex (F *versus M)***	0·95 (0·66, 1·36)	0·782			0·88 (0·59, 1·32)	0·531		
**Tumour site (oesophageal *versus g*astric)**	1·03 (0·75, 1·41)	0·853			0·64 (0·44, 0·94)	0·023	0·34 (0·23, 0·52)	< 0·001
**Neoadjuvant therapy (no *versus* yes)**	1·25 (0·91, 1·72)	0·170			2·17 (1·47, 3·20)	< 0·001		0·162
**Operation (THO *versus* TTO *versus* TG *versus* STG)**	1·00 (0·88, 1·15)	0·952			0·85 (0·72, 0·99)	0·039		0·296
**Individual surgeon (S1–S8)**	0·99 (0·93, 1·04)	0·620			1·02 (0·96, 1·08)	0·612		
**Team operating (no *versus* yes)**	0·96 (0·70, 1·32)	0·797			0·97 (0·68, 1·39)	0·875		
**Pathological factors**								
**T category (T1 *versus* T2 *versus* T3 *versus* T4)**	1·89 (1·60, 2·23)	< 0·001	1·66 (1·32, 2·07)	< 0·001	2·02 (1·66, 2·45)	< 0·001	1·82 (1·42, 2·33)	< 0·001
**N category (N0 *versus* N1 *versus* N2 *versus* N3)**	1·70 (1·49, 1·94)	< 0·001	1·34 (1·12, 1·60)	0·001	1·86 (1·59, 2·17)	< 0·001	1·59 (1·31, 1·94)	< 0·001
**TNM stage (I *versus* II *versus* III *versus* IV)**	2·10 (1·76, 2·50)	< 0·001		0·183	2·61 (2·10, 3·24)	< 0·001		0·688
**R status (R0 *versus* R1)**	2·30 (1·68, 3·14)	< 0·001		0·330	2·63 (1·84, 3·76)	< 0·001		0·896
**Postoperative factors**								
**Postoperative morbidity (no *versus* yes)**	1·41 (1·03, 1·93)	0·030	0·40 (0·20, 0·80)	0·527	1·20 (0·84, 1·71)	0·319		
**Clavien–Dindo morbidity grade**								
0	1·00 (reference)		1·00 (reference)		1·00 (reference)			
I	1·17 (0·58, 2·36)	0·653	1·20 (0·59, 2·43)	0·619	1·13 (0·51, 2·49)	0·759		
II	1·19 (0·81, 1·75)	0·382	1·52 (1·01, 2·29)	0·046	1,32 (0·88, 2·00)	0·182		
III	1·26 (0·76, 2·08)	0·375	1·70 (1·02, 2·86)	0·044	0·95 (0·52, 1·75)	0·880		
IV	1·67 (0·92, 2·02)	0·094	1·73 (0·94, 3·18)	0·077	1·31 (0·65, 2·65)	0·457		
V	380 (113·84, 1268·53)	< 0·001	198·27 (58·92, 667·17)	< 0·001	0 (0·00, 4·00 × 10^206^)	0·968		

Values in parentheses are 95 per cent confidence intervals. THO, transhiatal oesophagectomy; TTO, transthoracic oesophagectomy; TG, total gastrectomy; STG, subtotal gastrectomy; S, surgeon.

**Table 5:** Univariable and multivariable analysis of clinicopathological factors and complication markers, overall and disease‐free survival in patients with oesophageal cancer

	Overall survival				Disease‐free survival			
	Univariable analysis		Multivariable analysis		Univariable analysis		Multivariable analysis	
	Hazard ratio	*P*	Hazard ratio	*P*	Hazard ratio	*P*	Hazard ratio	*P*
**Age (< 65 *versus* 65–75 *versus* > 75 years)**	1·10 (0·78, 1·55)	0·581			0·92 (0·63, 1·35)	0·668		
**Sex (F *versus* M)**	1·13 (0·64, 2·00)	0·670			1·68 (0·84, 3·36)	0·142		
**Neoadjuvant therapy (no *versus* yes)**	1·62 (1·02, 2·57)	0·042		0·912	2·53 (1·46, 4·38)	0·001		0·362
**Operation (THO *versus* TTO)**	1·20 (0·84, 1·71)	0·309			1·02 (0·69, 1·50)	0·938		
**Individual surgeon (S1–S8)**	0·99 (0·92, 1·06)	0·733			0·97 (0·90, 1·05)	0·454		
**Team operating (no *versus* yes)**	0·98 (0·65, 1·47)	0·914			1·12 (0·73, 1·74)	0·598		
**Pathological factors**								
**T category (T1 *versus* T2 *versus* T3 *versus* T4)**	2·17 (1·70, 2·77)	< 0·001	1·60 (1·21, 2·10)	0·001	2·15 (1·67, 2·78)	< 0·001	1·70 (1·27, 2·27)	< 0·001
**N category (N0 *versus* N1 *versus* N2 *versus* N3)**	2·06 (1·72, 2·47)	< 0·001	1·66 (1·33, 2·07)	< 0·001	2·08 (1·69, 2·54)	< 0·001	1·61 (1·27, 2·05)	< 0·001
**TNM stage (I *versus* II *versus* III *versus* IV)**	2·35 (1·88, 2·94)	< 0·001		0·489	2·33 (1·84, 2·96)	< 0·001		0·607
**R status (R0 *versus* R1)**	3·31 (2·18, 5·02)	< 0·001		0·371	2·78 (1·79, 4·32)	< 0·001		0·792
**Lymph node yield (< 15 *versus* ≥ 15)**	1·16 (0·77, 1·75)	0·484			0·96 (0·67, 1·38)	0·822		
**Postoperative factors**								
**Postoperative morbidity (no *versus* yes)**	1·15 (0·76, 1·74)	0·499			1·04 (0·67, 1·61)	0·875		
**Clavien–Dindo morbidity grade**								
0	1·00 (reference)				1·00 (reference)			
I	1·00 (0·42, 2·36)	1·000			0·80 (0·28, 2·26)	0·797		
II	1·00 (0·62, 1·62)	1·000			1·10 (0·66, 1·82)	0·725		
III	1·00 (0·54, 1·85)	1·000			0·80 (0·39, 1·64)	0·546		
IV	1·00 (0·46, 2·17)	1·000			1·07 (0·49, 2·34)	0·861		
V	1·00 (0·00, 471·08)	1·000			0·00 (0·00, 127 × 10^195^)	0·966		

Values in parentheses are 95 per cent confidence intervals. THO, transhiatal oesophagectomy; TTO, transthoracic oesophagectomy; S, surgeon.

**Table 6:** Univariable and multivariable analysis of clinicopathological factors and complication markers, overall and disease‐free survival in patients with gastric cancer

	Overall survival				Disease free survival			
	Univariable analysis		Multivariable analysis		Univariable analysis		Multivariable analysis	
	Hazard ratio	*P*	Hazard ratio	*P*	Hazard ratio	*P*	Hazard ratio	*P*
**Age < 65 *versus* 65–75 *versus* > 75 years)**	1·22 (0·90, 1·66)	0·205			0·94 (0·64, 1·39)	0·764		
**Sex (F *versus* M)**	0·82 (0·50, 1·36)	0·446			0·35 (0·19, 0·66)	0·001	0·40 (0·21, 0·76)	0·005
**Neoadjuvant therapy (no *versus y*es)**	1·01 (0·61,1·67)	0·966			1·51 (0·81, 2·81)	0·194		
**Operation (TG *versus* STG)**	0·74 (0·49,1·11)	0·148			0·90 (0·53, 1·53)	0·700		
**Individual surgeon (S1–S8)**	0·99 (0·90,1·08)	0·794			1·09 (1·00, 1·20)	0·064		0·085
**Team operating (no *versus* yes)**	0·91 (0·55,1·49)	0·699			0·71 (0·37, 1·36)	0·304		
**Pathological factors**								
**T category (T1 *versus* T2 *versus* T3 *versus* T4)**	1·77 (1·37, 2·30)	< 0·001	1·81 (1·38, 2·37)	< 0·001	2·81 (1·84, 4·29)	< 0·001	2·12 (1·30, 3·47)	< 0·001
**N category (N0 *versus* N1 *versus* N2 *versus* N3)**	1·44 (1·17, 1·77)	0·001		0·768	2·14 (1·60, 2·87)	< 0·001	1·44 (1·01, 2·05)	0·043
**TNM stage (I *versus* II *versus* III *versus* IV)**	1·76 (1·31, 2·36)	< 0·001		0·484	3·41 (2·11, 5·51)	< 0·001		0·866
**R status (0 *versus* 1)**	1·36 (0·78, 2·40)	0·282			1·96 (0·99, 3·85)	0·052		0·658
**Lymph node yield (< 15 *versus* ≥ 15)**	0·82 (0·50, 1·34)	0·420			1·09 (0·57, 2·08)	0·803		
**Postoperative factors**								
**Postoperative morbidity (no *versus* yes)**	2·03 (1·24, 3·30)	0·005	0·28 (0·09, 0·89)	0·032	1·18 (0·62, 2·27)	0·612		
**Clavien–Dindo morbidity grade**								
0	1·00 (reference)				1·00 (reference)			
I	1·00 (0·32, 3·14)	1·000			1·70 (0·50, 5·57)	0·404		
II	1·00 (0·54, 1·85)	1·000			1·38 (0·66, 2·89)	0·399		
III	1·00 (0·40, 2·51)	1·000			0·96 (0·29, 3·20)	0·943		
IV	1·00 (0·20, 5·00)	1·000			1·15 (0·16, 8·56)	0·888		
V	1·00 (0·00, 566·54)	1·000			0·00 (0·00, 859 × 10^300^)	0·982		

Values in parentheses are 95 per cent confidence intervals. TG, total gastrectomy; STG, subtotal gastrectomy; S, surgeon.

## Discussion

This study examined compound‐level clinical outcome metrics across an UGI cancer network. The principal finding was that surgeon‐level annual data varied markedly. Operative mortality varied fivefold, anastomotic leak and overall morbidity twofold, lymph node harvest threefold and CRM positivity threefold. These annual variations resulted in a 5‐year cumulative OS rate that varied by nearly 50 per cent, and a 5‐year DFS rate that varied by about 30 per cent. Three‐year outcome measures demonstrated less variation than yearly measures and may be a superior reporting metric for surgical performance.

A consultant team‐focused operative approach to patients with a high‐risk profile was sevenfold safer in terms of operative mortality within 30 days. Over a 3‐year period, the operative mortality rate varied by 1·5 per cent, anastomotic leak by 2·2 per cent, overall morbidity by 13·9 per cent, lymph node harvest by 29 per cent, CRM positivity by 4·2 per cent, and 5‐year cumulative OS by 3·4 per cent. The hypothesis that there was no significant intersurgeon variation related to operative mortality was supported. Three‐year time frames provided a more balanced and uniform measure of performance than annual snapshots.

Clinical performance and patient outcome measures can be used to improve patient safety and clinical effectiveness. Public reporting of these metrics, such as individual surgeon outcome data, is designed to demonstrate transparency to consumers, allowing comparison and a sense of competition. For those involved in the organization and delivery of healthcare, these metrics can be used to set performance targets that may be associated with financial rewards or penalties at local, regional or national level.

In the context of UGI cancer surgery, operative mortality has been the outcome measure made publicly available in the UK^3^. The publication of mortality data as an indicator of quality of clinical care, however, may make some surgeons reluctant to operate on high‐risk patients^19^. Because surgical mortality rates are extremely low (about 2 per cent), one extra death has a notable impact on a surgeon's performance in a year, and risk‐adjustment methods cannot resolve such problems. The findings of this study suggest that 3‐yearly measures of quality including operative (margin status and lymph node yield) and postoperative information (Clavien–Dindo grade above II and postoperative death), together with 5‐year OS and DFS rates are necessary to measure surgical performance and outcome objectively.

Other UK centres have reported their experience after centralizing oesophagogastric cancer surgery^20^, ^21^, ^22^. In terms of compound‐level metrics, the reported rates for anastomotic leak were 7·3 per cent^20^ to 10·0 per cent^21^, margin involvement 46·0 per cent^20^, LOS 14 days^20^ and postoperative mortality between 0 and 3·6 per cent^20^, ^21^, ^22^. These figures are similar to those reported here and support the notion that higher patient volumes result in improved outcomes^23^. The centralization effect may in part reflect technical performance of the surgeon, but also includes performance of all team members contributing to perioperative care, the recognition and management of complications, and longer‐term nutritional support following discharge. Collectively, these features suggest that institutional data may be more useful to healthcare planners than those relating to individual surgeons.

This study has a number of limitations. Data were obtained from a single UK regional cancer network, so it is unclear to what extent the conclusions may apply elsewhere. Relatively few patients underwent a minimally invasive approach for either oesophageal or gastric cancer, as this was introduced in 2017. Conversely, data were collected in a contemporaneous way at all local and regional MDT meetings over a period of over 8 years; survival data were particularly robust because no patients were lost to follow‐up and death certification was obtained from the Office for National Statistics.

Improvements in any arena demand the measurement of results. Teams and their performance advance by tracing progress over time and relating performance to rivals both inside and outside their group. Rigorous value measurements (clinical outcomes and costs) are vital steps in refining healthcare. In the current arena of UGI cancer, where operative mortality is now low, this measure alone is no longer a robust predictor of long‐term survival nor a reliable measure of surgical performance when examined on an annual basis.

## Collaborators

Members of the South‐East Wales Oesophagogastric Cancer Collaborative: G. Blackshaw, G. Clark, A. Christian, X. Escofet, A. Foliaki, T. Havard, M. Henwood, J. Witherspoon and W. G. Lewis.

## Supporting information


**Table S1** Collective and individual surgeon‐level postoperative outcome
