# Supplementary material for: Value of individual surgeon performance metrics as quality assurance measures in oesophagogastric cancer surgery
Source: BJS Open. 2019 Nov 4;4(1):91–100. doi: 10.1002/bjs5.50230 (PMC6996630; doi:10.1002/bjs5.50230)
Supplement: Supplementary file 1 — Table S1 Collective and individual surgeon‐level postoperative outcome [file BJS5-4-91-s001.docx]

**BJS5_50230**

**Value of individual surgeon performance metrics as quality assurance measures in oesophagogastric cancer surgery**

**A. G. M. T. Powell, J. Wheat, N. Patel, D. Chan, A. Foliaki, S. A. Roberts and W. G. Lewis**

**Table S1** Collective and individual surgeon-level postoperative outcome

|  | Department | S1 | S2 | S3 | S4 | S5 | S6 | S7 |
| --- | --- | --- | --- | --- | --- | --- | --- | --- |
| Combined survival |  |  |  |  |  |  |  |  |
| Overall survival |  |  |  |  |  |  |  |  |
| 1 year | 92.1 | 97.7 | 92.4 | 90.4 | 90.8 | 87.8 | 96.2 | 90.6 |
| 2 year | 72.9 | 80.0 | 74.3 | 71.4 | 71.4 | 68.6 | 72.4 | 75.0 |
| 3 year | 62.2 | 63.6 | 62.5 | 63.2 | 58.2 | 60.6 | 61.1 | 70.0 |
| 5 year | 46.5 | 47.8 | 52.5 | 51.0 | 38.9 | 35.0 | N/A | N/A |
|  |  |  |  |  |  |  |  |  |
| Disease free survival |  |  |  |  |  |  |  |  |
| 1 year | 83.0 | 90.9 | 83.3 | 78.2 | 83.8 | 82.4 | 76.9 | 92.0 |
| 2 year | 67.0 | 71.4 | 66.7 | 63.1 | 70.4 | 65.6 | 53.3 | 84.6 |
| 3 year | 55.0 | 66.7 | 55.1 | 58.6 | 52.5 | 50.0 | 30.0 | 50.0 |
| 5 year | 44.8 | 47.1 | 60.0 | 44.4 | 35.7 | 28.6 | N/A | N/A |
|  |  |  |  |  |  |  |  |  |
| Oesophageal resection |  |  |  |  |  |  |  |  |
| Overall survival |  |  |  |  |  |  |  |  |
| 1 year | 93.7 | 100.0 | 92.0 | 94.9 | 88.9 | 96.2 | 97.2 | 86.4 |
| 2 year | 73.3 | 84.2 | 73.3 | 71.2 | 72.7 | 77.3 | 75.0 | 63.2 |
| 3 year | 62.5 | 61.1 | 66.7 | 60.0 | 60.7 | 71.4 | 55.6 | 53.8 |
| 5 year | 44.6 | 41.7 | 52.2 | 46.4 | 38.9 | 36.4 | N/A | N/A |
| Disease free survival |  |  |  |  |  |  |  |  |
| 1 year | 79.3 | 83.3 | 79.5 | 74.5 | 78.1 | 90.5 | 68.8 | 87.5 |
| 2 year | 62.3 | 58.8 | 64.1 | 52.4 | 70.4 | 70.0 | 50.0 | 77.8 |
| 3 year | 50.0 | 58.8 | 53.1 | 47.2 | 50.0 | 53.3 | 20.0 | 40.0 |
| 5 year | 37.2 | 30.0 | 55.6 | 36.0 | 29.4 | 25.0 | N/A | N/A |
|  |  |  |  |  |  |  |  |  |
| Gastric resection |  |  |  |  |  |  |  |  |
|  |  |  |  |  |  |  |  |  |
| Overall survival |  |  |  |  |  |  |  |  |
| 1 year | 89.6 | 94.4 | 93.1 | 82.9 | 92.5 | 73.3 | 94.1 | 100.0 |
| 2 year | 72.4 | 75.0 | 76.0 | 71.9 | 70.3 | 53.8 | 69.2 | 100.0 |
| 3 year | 61.8 | 66.7 | 54.5 | 67.7 | 55.6 | 41.7 | 66.7 | 100.0 |
| 5 year | 48.7 | 54.5 | 52.9 | 56.5 | 38.9 | 33.3 | N/A | N/A |
| Disease free survival |  |  |  |  |  |  |  |  |
| 1 year | 88.4 | 100.0 | 89.3 | 85.2 | 88.9 | 69.2 | 90.0 | 100.0 |
| 2 year | 74.3 | 90.9 | 71.4 | 82.6 | 70.4 | 58.3 | 57.1 | 100.0 |
| 3 year | 62.8 | 80.0 | 58.8 | 77.3 | 55.6 | 44.4 | 40.0 | 60.0 |
| 5 year | 57.4 | 71.4 | 66.7 | 63.6 | 45.5 | 33.3 | N/A | N/A |
